# Adult supratentorial extraventricular anaplastic ependymoma with cerebrospinal fluid dissemination metastases: a case report

**DOI:** 10.3389/fneur.2024.1351674

**Published:** 2024-02-28

**Authors:** Daojin Zhang, Hongbing Liu, Maosong Zhang, Jun Cao

**Affiliations:** Department of Neurosurgery, The First Affiliated Hospital of Wannan Medical College (Yijishan Hospital of Wannan Medical College), Wuhu, China

**Keywords:** supratentorial extraventricular, anaplastic ependymoma, dissemination metastases, operation, pathology

## Abstract

**Background:**

Ependymomas mostly locate in the infratentorial region and often occur in children. Anaplastic ependymomas account for 45–47% of supratentorial and 15–17% of infratentorial ependymomas, also known as malignant ependymomas. Adult supratentorial extraventricular anaplastic ependymoma (SEAE) is rare in clinical practice, and only a few cases have been reported so far, and there is no clinical study with large sample size. We report a case of adult supratentorial extraventricular anaplastic ependymoma in the occipital lobe with cerebrospinal fluid dissemination metastases.

**Case description:**

A 58-year-old female patient presented with unexplained pain in multiple parts of the body for the past half a year, mainly manifested as pain in the head, abdomen and chest. On August, 2022, Head MRI of the patient showed abnormal signal shadow in the left occipital lobe, which was considered a malignant lesion. The patient underwent tumor resection under general anesthesia on September 3, 2022. Postoperative pathological examination showed anaplastic ependymoma. The postoperative follow-up head MRI showed multiple cerebrospinal fluid dissemination metastases in the brain.

**Conclusion:**

Adult SEAE is a rare tumor with high malignancy and have a tendency to disseminate into the CSF, resulting in drop metastases. Immunohistochemistry is very important for the diagnosis of SEAE. It is recommended to administer adjuvant chemotherapy or radiation therapy appropriately after surgery, based on the tumor being completely resected as much as possible.

## Introduction

Ependymoma refers to central nervous system tumor originating from the ependymal cell of the ventricle and the central canal of spinal cord, or ependymal cell nest in the white matter of the brain. It often occurs in children and accounts for only 2–9% of adult neuroepithelial cell tumors (1). In 2021, World Health Organization (WHO) revised the classification of ependymoma to supratentorial ependymoma, posterior fossa ependymoma, spinal ependymoma, and subependymoma (2). As a WHO grade III tumor, supratentorial extraventricular anaplastic ependymoma has the characteristics of high recurrence rate, low survival rate and rare occurrence. At present, there are only a few case reports of supratentorial extraventricular anaplastic ependymoma, and there is no clinical study with large sample size. In this study, the clinical manifestations, imaging features, pathological features, treatment methods and prognosis of a patient with supratentorial extraventricular anaplastic ependymoma with cerebrospinal fluid dissemination metastases were analyzed and the results were reported as follows.

## Case presentation

**Figure 1:**
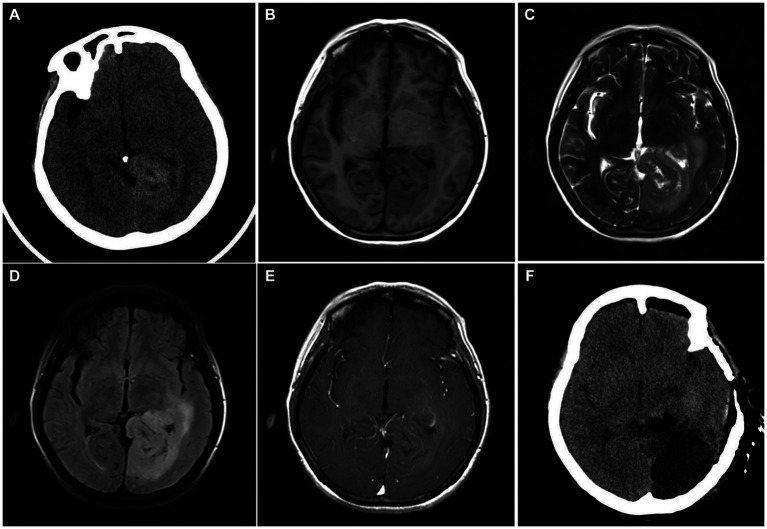
**(A)** Head CT on admission showed a slightly high-density mass in the left occipital lobe. **(B)** The lesion was hypointense on T1-weighted image. **(C)** T2-weighted image of head MRI showed hyperintensity of the lesion with surrounding edema. **(D)** T2-FLAIR image showed mixed signals with ill-defined borders. **(E)** Axial T1-enhanced image showed patchy mild enhancement of the lesion, part of the lesion encompassed the posterior part of the left lateral ventricle. **(F)** The head CT at 4 h after operation showed that the tumor was largely removed, and there was no obvious bleeding in the operation area.

A 58-year-old female patient presented with unexplained pain in the head, abdomen and chest in the past 6 months, and has been checked many times in the hospital without special abnormalities was found. Before admission, she experienced symptoms of nausea, vomiting, and fatigue, and has lost 10 kg of weight in the past 2 months. On August, 2022, PET-CT of the patient showed a hypermetabolic mass in the left occipital lobe and left basal ganglia, which was considered to be malignant. Visual examination upon admission revealed that the patient had contralateral hemianopsia in both eyes. After admission, the head CT showed a slightly high-density mass in the left occipital lobe, hippocampus, and corpus callosum. T2-weighted image of head MRI showed hyperintensity of the lesion with surrounding edema. Axial T1-enhanced image of head MRI showed patchy mild enhancement of the lesion, part of the lesion encompassed the posterior part of the left lateral ventricle (Figure 1).

On September, 2022, the patient underwent microsurgical resection of the space-occupying lesion in the left occipital lobe under general anesthesia. During the operation, the lesion can be seen to be dark red in color, with rich blood supply, infiltrative growth and soft texture. The lesion tissue and part of the occipital lobe were resected along the edema zone of the tumor. The size of the excised lesion is approximately 5.5 cm × 5.0 cm × 4.0 cm. Due to the extensive infiltration of the patient’s tumor, in order to preserve the patient’s neurological function and ensure life safety, the tumor was not completely removed during surgery.

**Figure 2:**
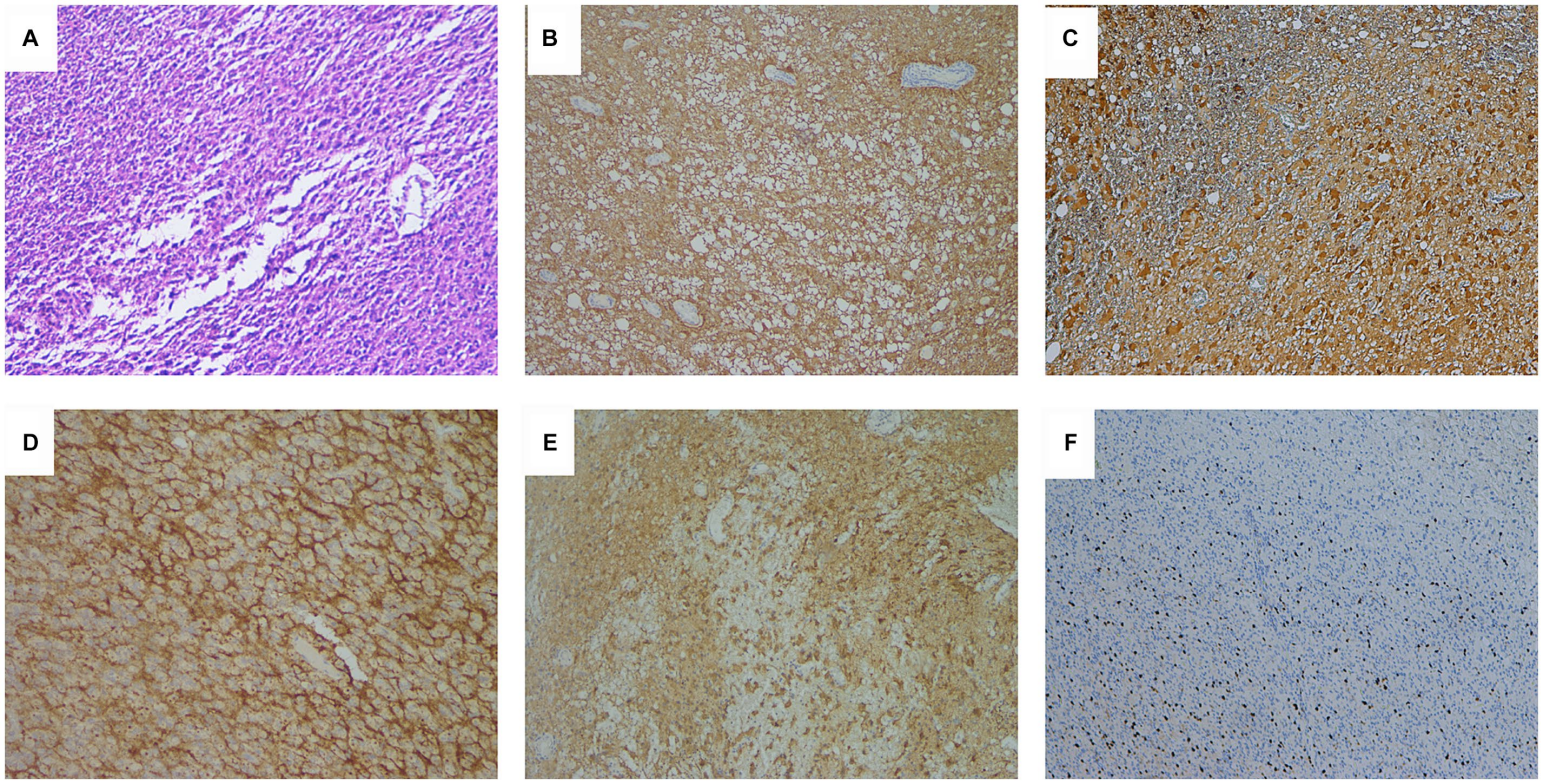
**(A)** Hematoxylin–eosin staining showed poorly differentiated tumor cells with hyperchromatic nuclei and proliferation of vascular endothelial cells (×100). **(B)** Immunohistochemical results showed diffuse cytoplasmic glial fibrillary acidic protein (GFAP) immunoreactivity (×100), **(C)** diffuse cytoplasmic and nuclear S100 immunoreactivity (×100), and **(D)** diffuse perinuclear epithelial membrane antigen (EMA) immunoreactivity suggestive of ependymal differentiation (×200). **(E)** Diffuse cytoplasmic and nuclear AE1/AE3 immunoreactivity (×100). **(F)** Ki-67 proliferation index was approximately 20% (×100).

**Figure 3:**
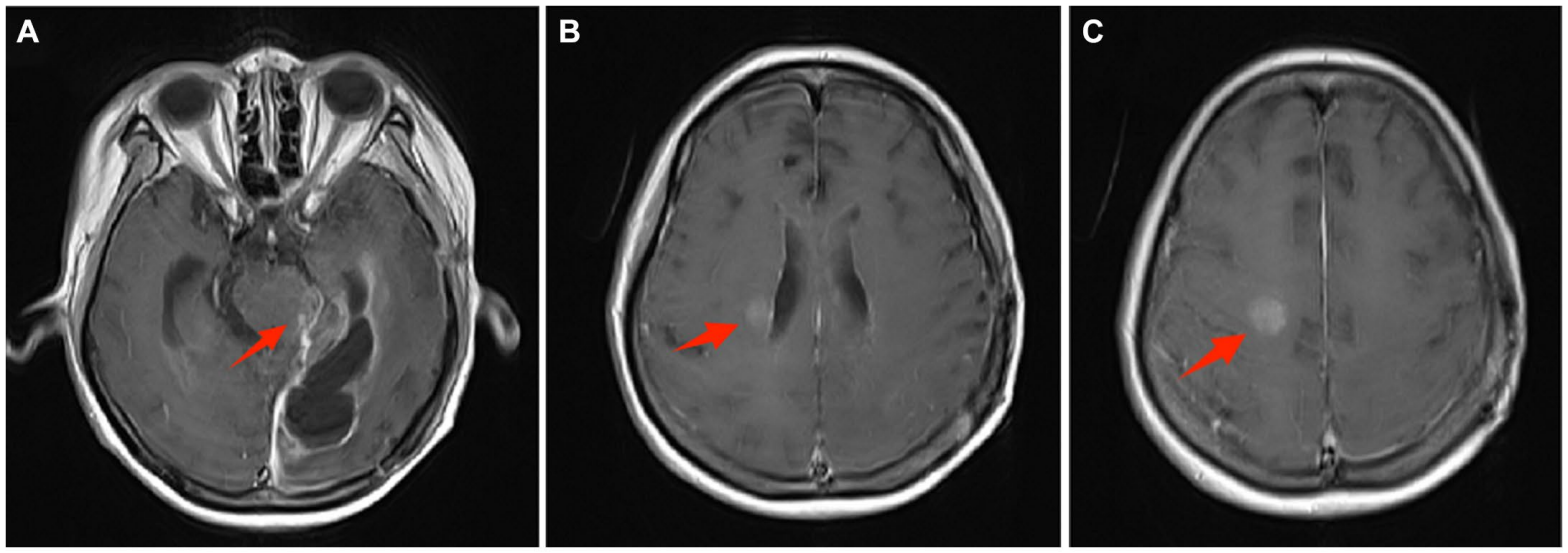
Eight months after operation, head MRI showed multiple cerebrospinal fluid dissemination metastases in the brain. **(A)** On enhanced magnetic resonance imaging, the arrow points to the tumor metastasis at the brainstem. **(B)** The arrow points to the metastatic tumor near the right ventricle. **(C)** The arrow points to a metastatic tumor in front of the right central sulcus.

The pathological diagnosis was anaplastic ependymoma (Figure 2). However, the patient’s consciousness gradually deteriorated and seizures occurred after operation. She was transferred to the neurosurgical intensive care unit and underwent tracheostomy 7 days after operation. After treatment, the patient regained clear consciousness, and had V-grade muscle strength in both limbs. One month after surgery, focal radiation therapy (59.40 Gy in 1.8 Gy daily fractions) and chemotherapy (temozolomide) were administered. Eight months after operation, the patient developed frequent epileptic seizures, and head MRI showed multiple cerebrospinal fluid dissemination metastases in the brain (Figure 3). The patient’s family decided to give up treatment, and the patient died 9 months after operation.

## Discussion

Supratentorial anaplastic ependymoma is a rare and highly malignant tumor, most of which occurs in the ventricle, but a small part of which can occur in the brain parenchyma and has no obvious correlation with the ventricle. Adult supratentorial extraventricular anaplastic ependymoma (SEAE) is rare in clinical practice, and only a few cases have been reported so far (Table 1). SEAE grows rapidly, mainly characterized by headache, vomiting, lethargy, anorexia, diplopia and other intracranial hypertension symptoms, and may be accompanied by epileptic seizures. Specific symptoms also depend on the location of the tumor, such as the tumor located in the frontal lobe, which can cause memory loss and mental abnormalities, and the tumor located in the occipital lobe, which can cause visual field changes (14). This patient presented with multiple unexplained pain throughout the body and visual field changes, which we speculated was related to the tumor invasion of the occipital lobe, the splenium of the corpus callosum and the hippocampus.

**Table 1:** Cases of supratentorial extraventricular anaplastic ependymoma in the literature.

Report	Age (years)	Sex	Location	Therapy	CSF dissemination metastases	Overall survival
Takeshima et al., 2002 (3)	70	Female	Right frontal lobe	Surgery	Not find	>14 months
Kojima et al., 2003 (4)	56	Female	Left temporal lobe	Surgery + radiotherapy	Not find	Unknown
Moritani et al., 2003 (5)	50	Female	Right temporal lobe	Surgery + chemotherapy	Not find	20 months
Miyazawa et al., 2007 (6)	33	Male	Left angular gyrus	Surgery + radiotherapy + chemotherapy	Not find	>6 months
Niazi et al., 2009 (7)	36	Female	Right frontal lobe	Surgery + radiotherapy	Not find	>29 months
	18	Male	Right frontal lobe			14 months
Davis et al., 2011 (8)	22	Female	Frontotemporal	Surgery + radiotherapy	Not find	>54 months
Romero et al., 2012 (9)	23	Male	Frontal lobe	Surgery + radiotherapy	Not find	Unknown
Iwamoto et al., 2014 (10)	61	Male	Right temporal lobe	Surgery + radiotherapy +chemotherapy	Dissemination metastases in spine	>12 months
Han et al., 2014 (11)	23	Male	Left occipital lobe	Surgery + radiotherapy	Dissemination metastases in spine	>48 months
Khilji et al., 2014 (12)	2	Male	Superolateral to the Right lateral ventricle	Surgery + radiotherapy	Not find	Unknown
Pachella et al., 2015 (13)	21	Male	Light temporoparietal	Surgery + radiotherapy + chemotherapy	Not find	Unknown
Lavrador et al., 2018 (14)	69	Female	Right frontoparietal	Surgery + chemotherapy	Not find	18 months
Kharosekar et al., 2018 (15)	11	Female	Right frontoparietal	Surgery + radiotherapy	Not find	Unknown
Seo et al., 2019 (16)	42	Male	Right frontal lobe	Surgery + radiotherapy	Not find	>12 months
Falcón et al., 2023 (17)	19	Male	Right frontal lobe	Surgery + radiotherapy	Not find	Unknown
This study	58	Female	Left occipital lobe	Surgery + radiotherapy + chemotherapy	Dissemination metastases in brain	9 months

The pathological features of anaplastic ependymoma were high tumor cell density, diverse cell morphology, poor cell differentiation, obvious nuclear atypia, more mitoses, and loss of the arrangement structure of ependymal epithelial cells (10). Compared with ependymoma, the perivascular pseudorosette structures of anaplastic ependymoma were significantly reduced, and vascular endothelial cell proliferation and pseudopalisade necrosis were common. Immunohistochemistry is very important for the diagnosis of anaplastic ependymoma. GFAP, S-100 and vimentin are positive in anaplastic ependymoma, and AE1/AE3 and EMA staining can also be positive (11).

Anaplastic ependymoma also have a tendency to disseminate into the cerebrospinal fluid (CSF), resulting in drop metastases (11). According to the 16 reported cases of supratentorial extraventricular anaplastic ependymoma so far, 3 of them reported cerebrospinal fluid dissemination and metastasis, with an incidence rate of 18.8% (Table 1). In the present case, dissemination and drop metastases occurred without recurrence at the primary site after adjuvant local field radiotherapy were identified.

The standard treatment for anaplastic ependymal tumors is total resection as far as possible, and adjuvant therapy such as appropriate local radiotherapy and chemotherapy after surgery (14). However, opinions on adjuvant therapy are divergent. For SEAE patients with high Ki-67 positive rate, some studies suggest that if the benefits of postoperative radiotherapy exceed the risk of recurrence of natural therapy in non-children and patients without obvious contraindications, appropriate radiotherapy can be given according to clinical practice (12). Therefore, the patient in this study was treated with appropriate extended resection of the lesion combined with local radiotherapy and chemotherapy.

According to previous literature, the factors that lead to poor prognosis of patients with anaplastic ependymoma include young age, incomplete tumor resection, tissue differentiation insufficiency, and tumor location above the tentorium and outside the ventricle. Among them, age and whether the tumor is completely resected are the most important factors (17). Ependymomas recur more frequently in the initial location after local failure. Kharosekar et al. (15) believed that if the tumor was completely located in the cortex, the patient might have a better prognosis. And, Saito et al. (16) reported that anaplastic ependymomas can disseminate within the central nervous system without local failure.

## Conclusion

Adult SEAE is a rare tumor with high malignancy and variable clinical features. SEAE also have a tendency to disseminate into the CSF, resulting in drop metastases. Immunohistochemistry is helpful for the diagnosis of SEAE. The treatment of adult SEAE has not been completely unified. It is recommended that on the basis of surgical treatment, appropriate adjuvant chemotherapy or radiotherapy should be given according to the specific conditions after surgery.

## Data availability statement

The datasets presented in this article are not readily available because of ethical and privacy restrictions. Requests to access the datasets should be directed to the corresponding author.

## Ethics statement

The studies involving humans were approved by the First Affiliated Hospital of Wannan Medical College (Yijishan Hospital of Wannan Medical College). The studies were conducted in accordance with the local legislation and institutional requirements. Written informed consent for participation in this study was provided by the participants’ legal guardians/next of kin. Written informed consent was obtained from the individual(s) for the publication of any potentially identifiable images or data included in this article.

## Author contributions

DZ: Resources, Validation, Writing – original draft. HL: Data curation, Formal analysis, Software, Supervision, Writing – original draft. MZ: Methodology, Project administration, Software, Supervision, Validation, Writing – original draft. JC: Conceptualization, Formal analysis, Funding acquisition, Supervision, Validation, Visualization, Writing – review & editing.
